# A study protocol of Older Person’s Exercise and Nutrition Study (OPEN) - a sit-to-stand activity combined with oral protein supplement – effects on physical function and independence: a cluster randomized clinical trial

**DOI:** 10.1186/s12877-018-0824-1

**Published:** 2018-06-07

**Authors:** Helena Grönstedt, Sofia Vikström, Tommy Cederholm, Erika Franzén, Åke Seiger, Anders Wimo, Gerd Faxén-Irving, Anne-Marie Boström

**Affiliations:** 1 Stockholms Sjukhem R&D unit, Stockholm, Sweden; 2 Allied Health Professionals, Function Area Occupational Therapy & Physiotherapy, Karolinska University Hospital, Stockholm, Sweden; 3 Department of Neurobiology, Care Science and Society, Division of Occupational Therapy, Karolinska Institutet, Stockholm, Sweden; 4 Department of Public Health and Caring Sciences, Clinical Nutrition and Metabolism, Uppsala University, Uppsala, Sweden; 5 Department of Geriatric Medicine, Uppsala University Hospital, Uppsala, Sweden; 6 Theme Aging, Karolinska University Hospital, Stockholm, Sweden; 7 Department of Neurobiology, Care Science and Society, Division of physiotherapy, Karolinska Institutet, Stockholm, Sweden; 8 Department of Neurobiology, Care Science and Society, Division of Clinical geriatrics, Karolinska Institutet, Stockholm, Sweden; 9 Department of Neurobiology, Care Science and Society, Division of neurogeriatrics, Karolinska Institutet, Stockholm, Sweden; 10 Allied Health Professionals, Function Area Clinical Nutrition, Karolinska University Hospital, Stockholm, Sweden; 11 Department of Neurobiology, Care science and Society Division of nursing, Karolinska Institutet, Stockholm, Sweden; 12 Western Norway University of Applied Sciences, Haugesund, Norway

**Keywords:** Interview, Mobility, Nursing home, Nursing staff, Nutrition, Older person, Oral nutritional supplement, Physical function, Quality of life, Sit-to-stand

## Abstract

**Background:**

Poor nutrition and age per see add to the development of sarcopenia, i.e. loss of muscle mass and strength, which contributes to increased risk of impaired activities of daily living (ADL) and reduced independence. Protein deficiency plays an important role in the development of sarcopenia. In order to increase the muscle mass protein intake should be combined with physical exercise. A daily physical activity, the sit-to-stand exercise, has been proven to decrease older persons’ dependence in ADL. Our study aims to evaluate the effects of the sit-to-stand exercise in combination with a protein-rich nutritional supplement, on physical function and independence in frail nursing home residents. The resident’s perceptions and experiences of the intervention and the staff’s experiences of supporting the resident to complete the intervention will also be explored.

**Methods:**

The study is a two-arm cluster-randomized controlled trial which will be performed in nursing homes at two municipalities in Sweden. We will recruit 120 residents, age 75 or older and able to stand up from a seated position. Residents (*n* = 60) randomized to the intervention group will perform the sit-to-stand exercise at four occasions daily and will be offered a protein-rich oral supplement, twice a day. The intervention period will last for 12 weeks and measures of physical function, nutritional status, quality of life and health economy will be performed at baseline and at 12-weeks follow-up. The primary outcome will be the number of chair rises performed in 30 s. The control group will receive standard care. Data will be analysed by intention-to-treat analysis and with mixed effect models. During the last part of the intervention period individual interviews with the residents, on the topic of feasibility with the OPEN concept will be held. Likewise, focus-group-interviews with staff will be performed.

**Discussion:**

The residents’ physical and mental health could be expected to improve. Even the work situation for staff could be positively affected. One innovative feature of the OPEN study is the simple intervention consisting of a basic daily activity that can be performed by several nursing home residents with the support of existing staff and available resources.

**Trial Registration:**

ClinicalTrials.gov Identifier: NCT02702037.

## Background

Active ageing is a concept introduced by World Health Organization in 2002 and defined as “*the process of optimizing opportunities for health, participation and security in order to enhance quality of life as people age”* [1]. Active ageing should be implemented for all older persons. For older residents living in nursing homes (NH), activities as meaningful leisure and participation, including physical activity, should be considered [2]. This is in line with the call for person-centered care where the focus is on the person’s expectations, resources, needs and abilities, and not on the symptoms and diseases. [3].

In spite of the knowledge of the health-related benefits of active aging and daily physical activity [2, 4], a majority of NH residents are inactive most of the time and with little social interaction [4–6]. This sedentary living is not necessarily due to medical conditions or decreased mobility function. The ward staff is often taking over the activities from the resident, in the meaning of being helpful, instead of letting the resident perform the activities, or part of activities, by themselves [7]. Mobility is identified by residents as a central importance to quality of life and well-being [8] and disability is one of the most consistent risk factors for depression in later life [9]. Inactivity and lack of social interaction can also lead to melancholy. Further, reduced activity and impaired mobility increases the risk of falls, pressure ulcers and incontinence [10, 11] all of which can lead to complications, hospitalization and reduced health-related quality of life [12]. Residents that are unable to move around independently, and even must rely on lifts for ambulation, often bears greater costs of care [13].

Aging causes loss of muscle mass and function which, along with poor nutrition contributes to increased risk of frailty. Sarcopenia, i.e., loss of muscle mass and muscle strength is probably the largest single cause of this scenario [14]. Sarcopenia often occurs in frail and sick older persons and leads to impaired ability to perform activities of daily living (ADL) such as walking, toileting, eating and socializing and thereby to increased dependence [12]. Protein deficiency seems to play an important role in the development of sarcopenia in older persons and for this reason the PROT-AGE study group as well as the New Nordic Recommendations recommend an increase in protein intake in older persons [15, 16]. In order to increase the muscle mass, the protein intake must be combined with physical exercise [17, 18].

It is possible to affect muscle strength with various forms of exercise, even in older NH residents. Progressive resistance training has been most studied [19]. High intensity group exercise program lead by physiotherapists has shown to improve balance control, strength and gait ability in frail older residents [20]. Similar to other studies [4], they investigated group training, distributed 3 times per week and on a relatively high level of intensity. However, there is scarce knowledge of the relation between the dose (frequency, duration, and intensity) of exercise in relation to the health outcomes. Frail older NH residents might benefit more by shorter exercise sessions several times a day rather than fewer and longer [21]. One functional and very essential exercise for older frail persons is standing up from a seated position and sitting down in a controlled manner - the sit-to-stand (STS) exercise. The ability to rise from a chair without help from others requires leg muscle strength and is fundamental for independent mobility. It is a simple activity, which as a physical exercise, does not require additional equipment and resources in the institutional setting. The exercise can be integrated with daily activities such as dressing, toileting or meals. The STS exercise has been studied in different settings with promising results [22–24]. A randomized-controlled trial study from Canada that evaluated the effects of STS exercise offered four times per day on seven days per week among older residents with dementia (mean age 88 years) living in long-term care facilities demonstrated less decline in mobility and functional outcomes in the intervention group compared with the control group [25].

In line with the above mentioned recommendations, a meta-analysis including 22 RCTs in both young and older subjects found that protein supplementation augments the adaptive response of skeletal muscle to resistance-type exercise training [17]. Protein supplementation improved physical performance and increased muscle mass gain during prolonged resistance-type exercise training in frail older people [18]. Moreover, a low protein intake in older persons in combination with low energy intake contributes to malnutrition and around 50% of older NH residents are assessed at risk of malnutrition [26]. For this reason, the OPEN-study aims to study the combination of exercise and protein supplementation on muscle mass and function.

Although there is evidence that STS exercise stabilizes older persons’ mobility, no studies are published exploring the older person’s experiences or perceptions of the STS exercise, neither on staff’s experiences on supporting older persons to complete the exercises. Interviews with older women with osteoporosis on their perceptions and experiences of physical training have revealed that personal preferences and individualization including support from health professionals was perceived as important [27]. Furthermore, there is a general lack of studies measuring the effectiveness of STS activities in this population. The novelty with the OPEN study is to evaluate the effects of the STS exercise during routine care, in combination with a protein-rich nutritional supplement, on functional status and independence in frail NH residents.

### Objectives

The main hypothesis of the OPEN study is that physical exercise performed as a daily routine along with a protein supplement, will result in improved physical function status and independence in everyday life activities and thereby enhance health-related quality of life in older persons living in nursing homes.

Specific aims of the study are:

- *Aim I:* To investigate the effects of the STS exercise combined with an oral protein-rich supplement on physical function and nutritional status, and health-related quality of life, and the frequency and incidence of falls, pressure ulcers and incontinence in older nursing home residents.
- *Aim II:* To estimate the cost effectiveness of the combination of the STS exercise and oral protein-rich supplement vs. the control group.
- *Aim III:* To describe the residents’ perceptions and experiences of daily being offered to conduct the STS exercise and to drink the nutritional supplement.
- *Aim VI:* To describe nursing home staff’s experiences of supporting the STS exercise and to offer the nutritional supplement to residents.

## Methods

### Trial design

**Fig. 1:**
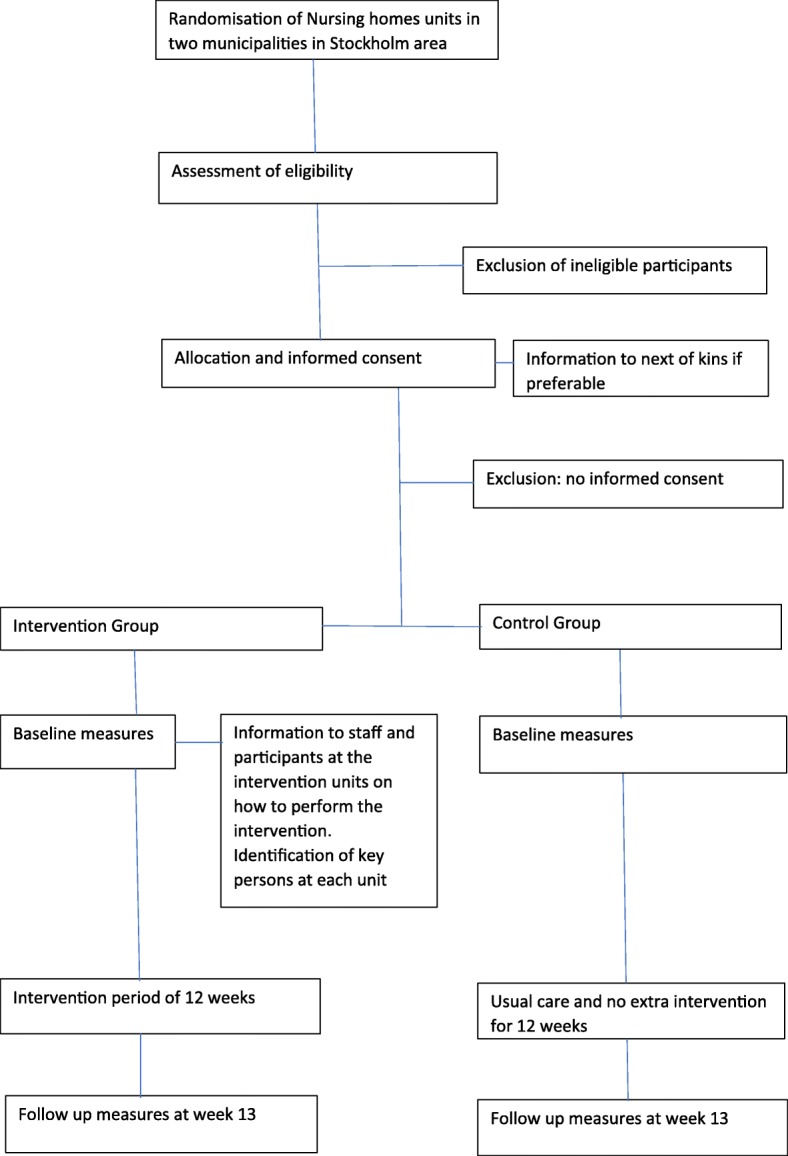
Design of the OPEN study

This study protocol has been informed by the SPIRIT guidelines [28]. The study is a two-arm cluster-randomized controlled trial which will be performed in NH at two municipalities in the Stockholm area. There are in total 62 NH units (34 dementia care, 28 somatic care; approx. 571 beds). A cluster will be defined as a group of 1–3 NH units with in total approximately 15–38 persons depending on the size of a unit. The clusters will be randomized according to a computer based sample list into two groups; intervention group and control group. The cluster design is chosen to prevent spread of the STS exercise to the control groups. This study has been registered with the number protocol of ClinicalTrials.gov Identifier: NCT02702037. An overview of the study is presented in Fig. 1.

### Ethics

The study has been approved by the Regional Ethical Review Board in Stockholm, D no. 2013/1659–31/2, 2015/1994–32 and 2016/1223–32. Major changes in the study protocol will be communicated to EPN for approval.

### Participants

Residents aged ≥75 years living in NH’s in the two municipalities will be invited to participate. Screening of all residents in the participating NH according to inclusion and exclusion criteria will be conducted by ward nurses and physiotherapists in cooperation with research staff. Eligible subjects will then be approached in person by the research staff for verbal informed consent. Information about the study will be given both in oral and written form.When needed, the research staff will read the written information loud to the older person.

#### Inclusion criteria

Able to rise from a seated position to standing.

#### Exclusion criteria

Persons with a Body Mass Index (BMI) > 30, treated with protein-rich oral supplement, suffering from severe dysphagia, tube fed, bedridden, severe kidney disease, at a terminal stage of life, lack of informed consent will be excluded. Resident who by the responsible nurse is assessed as not eligible to participate due to psychological or cognitive reasons will also be excluded. Psychological reasons is defined as anxiety and/or risk for increased behavioral problems due to something new added to the person’s daily life. Cognitive reasons is defined as not be able to follow instructions.

Among the older persons who have accepted participating in the intervention group, approximately 20 persons will be invited to participate in individual interviews. This is a number estimated to secure data saturation, which is strived for in Grounded theory methodology [29].

Staff, who will support the residents to complete the intervention, will be invited to participate in the qualitative part of the study. Staff will be recruited for interviews by convenience sampling, although with the criteria that they had experience of having supported residents with the intervention (STS and nutritional supplement).

### Intervention

#### Intervention group

The intervention period will last for 12 weeks and the participants in the intervention group will perform the STS exercise, where the older person will get up from a chair to stand and then sit down again repeatedly for 1–10 times at each session depending on the participant’s ability, at least four times per day, seven days a week. The exercise will take place in conjunction with daily activities for the older person such as dressing, toileting or at transfers, and be supported by trained staff. The staff will document in a flow chart how many times per day the participant completes the activity.

The staff will be educated about the objectives of the study, and the STS exercise, at group meetings at each unit. In cooperation with the unit manager, several “key-persons” in the staff will be identified, in order to be facilitators during the intervention. The physiotherapist, occupational therapist and/or nurse working at the unit will also be asked to encourage and give practical advice to the staff regarding the STS exercise. The key-persons and representatives of the research group will meet during the intervention period. The purpose for these meetings will be to encourage the key-persons and staff to discuss problems with the intervention that might occur.

The participants will also be offered an oral protein-rich supplement (125 ml, 18 g protein which is 24% of Recommended Dietary Intake, 300 kcal) twice a day (between breakfast and lunch and in the afternoon) during 12 weeks (7 days/week). Studies indicate that only between 50 and 65% of the prescribed volume is consumed (23). Therefore, we chose a supplement with low volume and high protein content. The drink will be served in specially designed study glasses, graded in quarters. The staff will register the intake of the supplements on a flow chart designed for this purpose.

Documentation of the daily exercises and nutritional supplements will be checked every week by the key-persons.

#### Harms

Solicited and spontaneously reported adverse events and other unintended effects of the trial interventions will be collected and reported by the ward nurses at the NH units.

#### Control group

The participants in the control group will receive standard care only and no extra intervention.

### Data collection/outcomes

The quantitative data will be collected at baseline and at the 12-week follow-up. An overview of the data collection is presented in Table 1. The following assessments will be performed to follow variables related to the primary and secondary aims for the study:

**Table 1:** Participant timeline

	Enrollment	Allocation	Postallocation		
TIMEPOINT	*-t*_*1*_	0	*t*_*1*_ *baseline*	*t*_*2*_ *intervention*	*t*_*3*_ *w 13*
ENROLLMENT:					
*Eligibility screen*	X				
*Informed consent*	X				
*Information to staff and next of kin*	X				
*Identification of* *“key-persons” and meetings together with representatives from the research group*		X	X	X	X
Allocation		X			
INTERVENTIONS:					
*Sit-to-stand*				X	
*Oral supplementation*				X	
ASSESSMENTS:					
*Sit-to-stand*			X		X
*Functional balance*			X		X
*Walking ability*			X		X
*ADL*			X		X
*Nutritional status*			X	X subgroup	X
*Health related quality of life*			X		X
*Healthcare resource use*			X		X
*Demographic data*			X		
*Medical incidences*				X	X
*Interviews of residents*					X
*Interviews of staff*				X	X

t_1_ baseline, t_2_ intervention period of 12 weeks, t_3_ 12 weeks follow up

#### Primary outcome

The number of chair rises in 30 s [30] will be the primary outcome for the study.

#### Secondary outcomes

##### Physical function

Functional balance will be measured by the Berg Balance scale [31, 32]. The scale consists of 14 tasks of relevance for everyday life and each item scored from 0 to 4, maximum score of 56. Walking speed will be measured (in participants who can walk) by walking propulsion indoors for a distance of 10 m at self-selected speed [33]. ADL will be measured with Functional Independence Measure which comprises 18 items grouped in the following functional domains: bathing, bowel management, toileting, eating, dressing lower body and social interaction [34, 35]. It is scored on a 7-point scale from 1 (dependent) to 7 (independent).

##### Nutritional status

Mini Nutritional Assessment-Short Form (MNA-SF) will be performed [36]. MNA-SF is scored 0–14 points. It includes six parts: evaluating decline in food intake, weight loss, mobility, physiological stress or acute disease, cognitive condition and BMI or calf circumference. 12–14 = normal nutritional status; 8–11 = at risk for malnutrition; 0–7 = malnourished. Food intake will be registered 2 consecutive days at baseline, at 6- and 12-week follow-up in a subgroup (25%) of the participants in the intervention group. Blood chemistry: Plasma albumin, plasma transthyretin, plasma C-reactive protein (CRP) to determine any active inflammatory process. Further the anabolic mediator plasma insuline-like growth factor-1 (IGF-I) and D-vitamin status will be assessed by S-25 (OH) vitamin D. Body composition measurements: Bioelectrical impedance analysis (BIA) [37].

*Quality of life* will be assessed using the EQ5D-5 L [38], a questionnaire comprising 5 questions corresponding to 5 health domains: pain, mood, mobility, self-care and daily activities. Each question has 5 levels: level one = no problems, level two, three and four = some, moderate or pronounced problems and level five = extreme problems. The participants’ opinion of their own health today, is valued according to a visual analogue scale. The scale is ranged from 0 = worst imaginable health state, to 100 = best imaginable health state.

*Healthcare Resource Use* data will be assessed by The Resource Utilization in Dementia (RUD©) [39] on the key cost components including: time of the healthcare assistant for personal ADL, hospitalizations (including length of stay), the number of visits to different types of healthcare professionals (both as outpatients and as a home visit), use of lifts.

##### Medical incidences

The effects of the intervention on frequency and incidence of falls, pressure ulcers and incontinence will be assessed from patient records and study documents at baseline and at the 12-weeks follow-up. Falls: Falls documented by health care professionals in patient records will be monitored to assess the safety of the intervention and the effects on incidence of falls. Pressure ulcers: Information on the occurrence of pressure ulcers will be obtained from the patient health record. Mobility related to toilet habits: Mobility status related to toilet habits will be recorded; whether the participant is self-reliant; needs supervision or some help; or is totally dependent on help. Information on this data will be recorded in a specific document for the study.

#### Sample characterization

*Demographic data* on age, sex, and medical diagnosis will be collected and described. *Cognitive function* will be assessed using Mini Mental State Examination (MMSE; 0-30p) [40]. *Sarcopenia* will be assessed according to the recommendation of EWGSOP [14] by SARC-F questionnaire [41] and *frailty* will be assessed by the Frail questionnaire screening tool [42].

### Sample size calculation

The sample size calculation for the number of participants is based on the effect size of the primary outcome measure in a pilot study performed and described in a study protocol by a Canadian research group that we collaborate with [24]: that is the number of sit-to-stands that the participant is able to complete in 30 s. An increase of 2 sit-to-stands completed in 30 s is suggested to be a meaningful change in the person’s mobility. Based on existing research [30, 43–45] the standard deviation of the 30-s sit-to-stand measure is 3.5 sit-to-stands. Based on the power calculation by Slaugther and colleagues [24], 49 participants in each group are needed to detect an increase in the number of sit-to-stands (power = 0.80; 2-tailed; α = 0.05). Assuming an attrition of 20% over the 12 weeks, we will recruit in total 120 participants (60 participants to each group).

### Data collection/interviews

To collect data for the third and fourth aims, interviews will be conducted in the middle of the intervention (at 6–8 weeks) and at the 12-weeks follow-up.

The interviews of the residents will be collected in a secluded locality in the participants´ homes within the NH. Each person interviewed will be asked to describe how he/she perceived the experience daily being offered to conduct the STS exercise and to drink the nutritional supplement (with support of staff). A special focus will be held on questions concerning health, wellbeing and independence. Interviewing persons with memory impairment has been suggested to be potentially problematic [46].

A common problem is that persons with dementia might have difficulties recalling answers to specific questions and staying on subject. Furthermore, a common argument is that the memory loss is not static, which means that the person with dementia sometimes remembers more aspects on a topic than at other times [47]. Inclusion of persons with dementia in interviews is suggested feasible although the memory can fail in some detail. For example, Wikström [48] proved this to be a fruitful method, finding that a complex picture of the interviewees’ perceptions became visible. To discover each person’s unique, individual descriptions, they suggest to interview the participants separately. From an ethical viewpoint, this also seemed a fair approach for all older participants, since they in that way are equally acknowledged by the researcher.

In a similar vein to the interview questions for the residents above, interviews on the same topic will be posed to staff in the NH’s. Here, a focus group methodology will be used [49, 50]. The strength of a focus group methodology is that the group is viewed to facilitate and enable respondents to share experiences, that in turn will feed in to- or trigger- the memories of others, that will be able to share more in-depth thoughts and ideas. The interviews will be conducted in groups of 3–5 staff, and will be tape-recorded [51, 52]. The interviewees will describe their perceptions of promoting the STS exercise and the intake of the nutritional supplement with the NH residents, including e.g. experienced barriers and strategies to overcome them, but also on the residents’ health, wellbeing and independence.

The interviewer will not interrupt unless the interviewee indicated that he/she had finished elaborating on a topic, or if a follow-up question was of interest to reach further depth in the descriptions. Sometimes, however, the participants might need to be reminded of the researcher’s focus of interest, which will be done by interruption and a repetition of the question. The time the participants will use to describe their perceptions and experiences of the OPEN project on their health and wellbeing will approximately range from 20 to 50 min.

Interview guides for the individual interviews and the focus group interviews will be developed. Field notes on aspects that might affect the perceptions of the sit-to stand activity told either by the participants themselves or the staff will be written down in immediate conjunction to the interviews [53].

### Data collection procedure

When a resident has accepted to participate, the data collection will be performed by two clinically experienced physiotherapists from the research group at baseline and at follow-up. The data will be collected at the participating NH’s. Assessments will take place in the participant’s room and in the facilities outside the room (i.e. the corridor for the walking test). The assessors will discuss the tests before study start to ensure high reliability of assessments. Blood samples will be collected and handled by 2 persons experienced in collecting and handling blood samples not routinely used in nursing homes. Demographic data will be retrieved from patient records with access help from the ward nurses.

Interviews with staff and NH residents will be performed by a researcher with great experience of qualitative research and interviewing older persons and staff.

Enrolment to the study will be ongoing until 120 participants, 60 in each group, are included.

### Blinding

The blinding of assessors was determined to be challenging to achieve due to the cluster design. Clues in the environment (lists, reminders, bottles) and risk of accidental information given by participants and/or staff could easily reveal the unit’s group assignment.

### Data management and monitoring

The participants will be given a unique code for identification. All data will be stored in a folder for each participant and the folder will be kept in a locked cabinet at each nursing home during study time. When the study is concluded, or the participant’s drops out, each folder will be stored according to the routines for archives of research material. After baseline and follow up assessments, the data will be registered in SPSS files at a lap-top especially designated for the OPEN study. Back up files will be created and stored. The interviews will be tape-recorded, transcribed and stored according to the routines for archives of research material.

The study is initiated and planned by the research study group with a multi-professional composition (physicians, physiotherapists, occupational therapist, nurse and dietitian).

No interim analyses of the data will be performed. The research group will have access to the final dataset. Each member of the research group will be allowed to share some of the data supervising master or PhD-students who will be involved in the analyses.

### Data analyses

All data will be entered into a database using SPSS (iBM). Quality controls will be made to check that the data are entered correctly. The baseline characteristics of the participants in the intervention and control groups will be compared using descriptive statistics. All analysis will be done by members of the research group and by an independent statistician respectively.

Data for primary and secondary outcome measures will be analysed using descriptive and comparative statistical analyses including multi-level statistics appropriate for the data measurement levels and data distributions. Data will be analysed according to intention-to-treat, i.e. participants who will drop out before completing the 12 weeks of follow up will be included in the data analysis. The model of data analysis needs to consider the effect of clustering. Thus, this study will use mixed effect models with the cluster treated as a random effect.

Combining the resource units with the cost price will allow the calculation of total costs of each group. The cost effectiveness calculation will use the EQ5D-5 L as outcome. Costs and effects will be examined based on the principle of cost-consequence analysis. This type of analysis lists all relevant costs and effects (consequences) in order to capture as much information as is feasible, in order to allow decision makers to choose the outcome of interest for inclusion in economic analyses [54].

The qualitative data consisting of tape-recorded interview-answers will be transcribed verbatim. The qualitative data sets will be analysed from a data-driven inductive approach. More specifically, the transcripts of the interviews will be read and analysed using a constant comparative method described by [29].

The analysis of the qualitative data will have a Grounded theory (GT) approach. Traditionally, GT has been applied when the ambition to capture phenomena in different social contexts have been in focus. GT is also used as a reliable technique to form theory and hypothesis in un-exploited areas. The findings from a GT study often entail facts that are valuable assets in development of assessments, creation of questionnaires and so forth in quantitative studies [29]. According to GT methodology, the analysis is performed in different steps. Initially, the interview transcripts are read through repeatedly until the researcher has a full understanding of them. Thereafter a so called line-by-line coding of the material is performed [55]. Here, significant aspects that relate to the aim of the study, and the specific foci of the research questions are marked and saved. Thereafter, the codes are sorted in a structured manner to identify trends and patterns so that they can be collected under different clusters; called categories [50]. In the final stage, the material is scrutinized from the different categories’ viewpoint to identify and let potential abstracted themes to emerge. As the analysis undergo a constant comparison process, securing that the aim of the study is held in the fore through all analytic steps, the trustworthiness of the codes, categories and themes respectively, are to be considered high [55].

### Dissemination policy

Trial results will be communicated by the research group to the participating NH’s (managers, staff, residents, family members and relatives) and to the sponsors, the healthcare professionals and the public. The study will result in several different publications in peer reviewed journals.

## Discussion

### The intervention

The STS exercise has earlier been studied in different settings with promising results. The novelty with this study design is the combination of the STS exercise with protein-rich supplements. The study by Slaugther and colleagues [25] revealed that the low-intensive STS exercise for 6 months could be performed by older NH residents, and that the participants in the intervention group, to a greater extent, maintained their mobility and functional outcomes compared with the control group. If the STS exercise together with a protein-rich oral supplement results in improved muscle strength in older frail persons, it would increase their functional status and lead to greater independence in activities of daily living. The residents’ physical and mental health could be expected to improve. Even the work situation for staff would be positively affected. One innovative feature of the OPEN study is the simple intervention consisting of a basic daily activity that can be completed by several of NH residents with the support of the existing staff and actual resources. It would also be of interest if a positive result can be obtained in 3 months of intervention compared to the 6 months of intervention in the study by Slaughter et al. [25]. A positive result of our study could be important for many NH residents for the purpose to postpone physical decline and decreased independence.

A crucial point of the study design will be to educate and motivate the staff to support the residents to complete the intervention, i.e. the STS exercise and intake of oral nutritional supplement. To meet these challenges, “key-persons” at each unit in the staff will be identified and educated. The physiotherapist, occupational therapist and nurse working at the unit will also be asked to encourage and give practical advice to the staff regarding the STS exercise.

### Data collection procedure

Residents living in Swedish NH’s today are frail and vulnerable and a rapid decrease in length of stay between 2006 and 2012 has been reported. The most dramatic change was an increase in the proportion of people who moved into an institution and died shortly afterwards [56]. This will probably result in a prolonged recruitment process to the study. A pilot study was performed at one NH with 81 residents to test the study protocol and its feasibility. Some changes were made in the protocol regarding exclusion criteria. The cut off for BMI was raised from 28 to ≤30 and Mini Mental State Examination will be used [40] to assess and describe cognitive function instead of Short Portable Mental Status Questionnaire [57].

### The interviews (staff, residents)

Although there is evidence that STS exercise stabilizes older residents’ mobility no studies are, to our knowledge, published exploring the residents’ experiences or perceptions of the STS exercise, neither on staff’s experiences on supporting older persons to complete the exercises. A strength of the focus group methodology, which will be used in the interviews with the staff, is that it enables respondents to share experiences, that in turn will feed into- or trigger- the memories of others, that will be able to share more in-depth thoughts and ideas. The interviews of the residents how he/she perceived the experience of daily being offered to conduct the STS exercise and to drink the nutritional supplement (with support of staff) will be useful knowledge and valuable information for future studies.

### Healthcare resource use

Another strength of the study design is the *Healthcare Resource Use* data that will be assessed by The Resource Utilization in Dementia (RUD©) [39] on the key cost components; time of the healthcare assistant for personal ADL, hospitalizations (including length of stay), the number of visits to different types of healthcare professionals (both as outpatients and as a home visit), use of lifts. Economic aspects are important to include in intervention studies like this to encourage implementation of new routines.

In conclusion, the OPEN study design with a combination of daily basic activity and protein rich oral supplement in NH residents will add new knowledge in several aspects in this field. The effects of the multicomponent intervention regarding mobility/functional status, nutritional status and health economy will be highlighted. The presumed simplicity of the intervention will also be explored by staff and resident’s experiences.

## Abbreviations

ADL: Activity daily living
BMI: Body mass index
GT: Grounded theory
MNA-SF: Mini Nutritional Assessment – Short Form
NH: Nursing home
OPEN: Older person’s exercise and nutrition study
RUD: The Resource Utilization in Dementia
STS: Sit to stand
